# The needs of members of the families of general hospital inpatients

**DOI:** 10.1590/S1516-31802008000200013

**Published:** 2008-03-06

**Authors:** Ana Cecília Lucchese, Vanessa de Albuquerque Citero, Mario Alfredo De Marco, Sergio Baxter Andreoli, Luiz Antonio Nogueira-Martins

**Keywords:** Hospitals, general, Caregivers, Needs assessment, Inpatients, Mental disorders, Hospitais gerais, Cuidadores, Determinação de necessidades de cuidados de saúde, Pacientes internados, Transtornos mentais

## Abstract

**CONTEXT AND OBJECTIVE:**

The needs of members of the families of intensive care unit patients have been studied, but little is known about the needs of members of the families of general hospital inpatients, especially patients with chronic diseases. The aim of this study was to identify the needs of members of the families of general hospital inpatients and investigate associations between these needs and the patients’ clinical and psychiatric profiles.

**DESIGN AND SETTING:**

Descriptive study, in a public teaching hospital.

**METHODS:**

A random sample of 47 patients and members of their families was studied. Family members’ needs were investigated using the critical care family needs inventory and the patients’ clinical profiles were investigated using the hospital anxiety and depression scale, confusion assessment method and Karnofsky performance status. The frequencies of family members’ needs were described and the patients’ clinical and psychiatric characteristics were correlated with the needs using the chi-squared test.

**RESULTS:**

Chronic patients predominated and the needs for reassurance and information were indicated as the most important by all members of their families. No associations were found between the patients’ characteristics and the needs of members of their families during the hospitalization.

**CONCLUSIONS:**

The needs indicated by members of the families of general hospital inpatients were similar to those of members of the families of patients in intensive care units: they considered it very important to be reassured and kept informed throughout the hospitalization.

## RESUMO

**CONTEXTO E OBJETIVO:**

As necessidades dos familiares de pacientes internados têm sido estudadas em unidades de terapia intensiva, porém pouco se conhece sobre as necessidades dos familiares de pacientes internados no hospital geral, especialmente dos pacientes com doenças crônicas. O objetivo deste estudo foi identificar as necessidades dos familiares de pacientes internados no hospital geral e verificar associações dessas necessidades com o perfil clínico e psiquiátrico dos pacientes.

**TIPO DE ESTUDO E LOCAL:**

Estudo descritivo em hospital-escola público.

**MÉTODO:**

Numa amostra aleatória de 47 pacientes e seus familiares, as necessidades dos familiares foram investigadas com o uso da CCFNI (Critical Care Family Needs Inventory); o perfil clínico dos pacientes com os instrumentos: HAD (Hospital Anxiety and Depression Scale), CAM (Confusion Assessment Method) e KPS (Karnofsky Performance Status). Foram descritas as freqüências das necessidades dos familiares; a associação das características clínicas e psiquiátricas às necessidades foi analisada com o uso de teste qui-quadrado.

**RESULTADOS:**

Houve predominância de pacientes crônicos e as necessidades de segurança e informação foram apontadas como as mais importantes por todos os familiares. Não foram encontradas associações entre as características dos pacientes e as necessidades dos familiares durante a hospitalização.

**CONCLUSÕES:**

As necessidades apontadas pelos familiares dos pacientes internados no hospital geral foram similares às que são apontadas por familiares de pacientes internados em unidades de terapia intensiva: eles consideraram muito importante sentirem-se assegurados e informados durante todo o período de internação.

## INTRODUCTION

Any hospitalization represents a moment of crisis for patients and their families.^1^ Members of patients’ families tend not to cooperate with medical teams, and special care needs to be taken so that family members are able to identify themselves in this situation and can receive and understand important information without impedance and without interfering with the patients’ treatment and hospital routines and procedures.^2^ The presence of members of patients’ families during hospitalization can offer countless benefits for medical treatment: it has been observed they have a positive impact on inpatients, acting as "tranquillizers" and placating their anxieties.^2^ Consequently, understanding the needs of members of patients’ families during hospitalization is important, as is knowing what it is that determines such needs. This issue has been studied among members of the families of patients in intensive care units^3^ but very little is known about members of the families of patients hospitalized in wards at general hospitals, and especially chronic patients.

## OBJECTIVE

This study had the aim of identifying the needs of members of the families of inpatients in a Brazilian general hospital and assessing the association of such needs with the clinical and psychopathological profiles of hospitalized patients. Our hypothesis was that members of the families of inpatients in a clinically more severe condition or presenting psychiatric comorbidity had needs that differed from those of members of the families of patients who were in a clinically less severe condition or who did not have psychiatric comorbidities.

## METHOD

This was a cross-sectional study at Universidade Federal de São Paulo — Escola Paulista de Medicina (Unifesp-EPM). A random sample of 47 beds that were managed within the national health system wards was created. Each bed represented a set comprising a patient, members of his/her family, a physician and a nurse. In this present paper, we consider only patients’ and family members’ responses; other results and a detailed description of the methods have been published previously.^4^

Patients were included if they were more than 18 years old and hospitalized for a minimum of 24 hours (not in an intensive care unit) and if they had ability to respond to the questionnaires. The clinical and demographic data on patients who were excluded were recorded for statistical control of losses. The family members included were the ones mentioned by each patient.

The patients filled out clinical and sociodemographic questionnaires and, to evaluate the possible presence of psychiatric comorbidities, they also gave responses in relation to the Brazilian validation of the hospital anxiety and depression scale (HAD) and the Brazilian validation of the confusion assessment method (responses noted down by the interviewer). Simultaneously, another interviewer applied the Karnofsky performance status (KPS) measurement to evaluate the level of the patients’ dependence due to their physical disease.

Members of patients’ families filled out a sociodemographic questionnaire, an information questionnaire and the Brazilian validation of the critical care family needs inventory (CCFNI). The latter contains 43 questions about importance of five categories of needs: information, reassurance, accessibility, support and comfort.

The frequencies and means of all the data were examined. The degree of importance of each need was correlated with the patients’ sociodemographic, clinical and psychiatric characteristics, and with the family members’ sociodemographic characteristics, kinship, and information level, using the chi-squared test and Fisher’s exact test when appropriate.

## RESULTS

More than half of the patients were more than 45 years old and they had been ill for more than one year, principally due to neoplasia, with previous hospitalizations (Table 1). 33% presented greater dependence due to their physical disease, 35.7% had depressive symptoms and 45.2% had anxiety symptoms. The members of patients’ families were predominantly female; they visited the patients frequently, shared close ties with them and received help from other people when caring for the patients (Table 2). In general, they were well informed about the patients’ disease and its duration, but only one third of the family members could identify the physician who was responsible for the patient and only 19% the corresponding nurse.

**Table 1:** Distribution of the sociodemographic, clinical and psychiatric characteristics of inpatients, according to gender. p ≥ 0.05 for all variables

		Male	Female	Total
**Age**	18 to 30 years	4 (21.1)	2 (7.1)	6 (12.8)
	31 to 45 years	4 (21.1)	9 (32.1)	13 (27.2)
	46 to 65 years	9 (47.4)	8 (28.6)	17 (36.2)
	Over 65 years	2 (10.5)	9 (32.1)	11 (23.4)
**Schooling**	0 to 4 years	7 (36.8)	6 (21.4)	13 (27.2)
	5 to 8 years	6 (31.6)	10 (35.7)	16 (34)
	9 or more years	6 (31.6)	12 (42.9)	18 (38.3)
**Economically active**	Yes	12 (63.2)	6 (21.4)	18 (38.3)
	No	7 (36.8)	22 (78.6)	29 (61.7)
**Duration of disease**	0 to 6 months	5 (26.3)	10 (35.7)	15 (31.9)
	7 to 12 months	4 (21.1)	4 (14.3)	8 (17)
	More than 12 months	10 (52.6)	14 (50)	24 (51.1)
**Hospitalization diagnosis**	Neoplasia	4 (21.4)	8 (28.6)	12 (25.5)
	Cardiovascular diseases	4 (21.4)	4 (14.3)	8 (17)
	Osteomuscular system	4 (21.4)	1 (3.6)	5 (10.6)
	Other diseases	7 (36.8)	15 (53.6)	22 (46.8)
**Previous hospitalizations**	No	12 (63.2)	11 (39.3)	23 (48.9)
	Yes	7 (36.8)	17 (59.7)	24 (51.1)
	**Total**	**19 (100)**	**28 (100)**	**47 (100)**
**Physical dependence**	Lower (KPS = 90 to 80)	3 (16.7)	7 (25.9)	10 (22.2)
	Moderate (KPS = 70 to 50)	8 (44.4)	12 (44.4)	20 (44.4)
	Greater (KPS = 40 to 20)	7 (36.8)	8 (29.6)	15 (33.3)
	**Total**	**18 (100)**	**27 (100)**	**45 (100)**
**Depressive symptoms**	Yes (HAD ≥ 9)	2 (11.8)	13 (52)	15 (35.7)
	No (HAD ≤ 8)	15 (88.2)	12 (48)	27 (64.3)
**Anxiety symptoms**	Yes (HAD ≥ 9)	5 (29.4)	14 (56)	19 (45.2)
	No (HAD ≤ 8)	12 (70.6)	11 (44)	23 (54.8)
	**Total**	**17 (100)**	**25 (100)**	**42 (100)**
**Delirium**	Yes		3 (10.7)	3 (6.7)
	No	17 (100)	25 (89.3)	42 (93.3)
	**Total**	**17 (100)**	**28 (100)**	**45 (100)**

*KPS = Karnofsky Performance Status; HAD = Hospital Anxiety and Depression Scale.*

**Table 2:** Distribution of the sociodemographic characteristics, kinship and level of information among members of inpatients’ families, according to gender. p ≥ 0.05 for all variables

		Malen = 11 (100%)	Femalen = 36 (100%)	Totaln = 47 (100%)
**Sociodemographic characteristics**					
Age	18 to 30 years	3 (27.3)	12 (33.3)	15 (31.9)
	31 to 45 years	3 (27.3)	5 (13.9)	8 (17)
	46 to 65 years	5 (45.5)	16 (44.4)	21 (44.7)
	Over 65 years		3 (8.3)	3 (6.4)
Schooling	0 to 4 years	1 (9.1)	6 (16.7)	7 (14.9)
	5 to 8 years	4 (36.4)	10 (27.8)	14 (29.8)
	9 or more years	6 (54.5)	20 (55.6)	26 (55.3)
Economically active	Yes	6 (54.5)	9 (25)	15 (31.9)
	No	5 (45.5)	27 (75)	32 (68.1)
**Kinship characteristics**					
Degree of kinship	Parents		4 (11.1)	4 (8.5)
	Companion	2 (18.2)	14 (38.9)	16 (34)
	Sibling	2 (18.2)	5 (13.9)	7 (14.9)
	Son/Daughter	5 (45.4)	6 (16.7)	11 (23.4)
	Other	2 (18.2)	7 (16.7)	9 (19.7)
Does this family member live with the patient?	Yes	8 (72.7)	25 (69.4)	33 (70.2)
	No	3 (27.3)	11 (30.6)	14 (29.8)
Has this family member ever accompanied the patient previously?	Yes	6 (54.5)	22 (61.1)	28 (59.6)
	No	5 (45.5)	14 (38.9)	19 (40.4)
Is there another member of the family who helps to care for the patient?	Yes	8 (72.7)	24 (66.7)	32 (68.1)
	No	3 (27.3)	12 (33.3)	15 (31.9)
Frequency of visits to patient per week	1 to 4 days	4 (36.4)	16 (44.4)	20 (42.6)
	5 to 7 days	7 (63.6)	20 (55.6)	27 (57.4)
**Information questionnaire**					
Does the family member know the patient’s diagnosis?	Yes	5 (45.5)	27 (75)	32 (68.1)
	No	6 (54.5)	9 (25)	15 (31.9)
Does the family member know how long the patient has had the disease?	Yes	6 (54.5)	20 (55.6)	26 (55.3)
	No	5 (45.5)	16 (44.4)	21 (44.7)
Does the family member know the name of the physician in charge?	Yes	3 (27.3)	15 (41.7)	18 (38.3)
	No	8 (72.7)	21 (58.3)	29 (61.7)
Does the family member know the name of the nurse in charge?	Yes	1 (9.1)	2 (22.2)	9 (19.1)
	No	10 (90.9)	28 (77.8)	38 (80.9)
**Total**		**11 (100)**	**36 (100)**	**47 (100)**

Thirteen of the 43 questions in the CCFNI were considered by all members of families as being "very important" (Table 3). We did not find any statistically significant association between the patients’ clinical and psychiatric variables and the needs of members of their families. The questions referring to reassurance were the ones deemed most important among our sample. Questions relating to information also stood out. Only two questions relating to accessibility were among the most important: one relating to comfort and one to support.

**Table 3:** Questions from the critical care family needs inventory that were classified as being of great importance by all members of inpatients’ families

Questions	Category
Knowing what the patient’s chances of improvement are.	Reassurance
Having questions answered openly and honestly.	Reassurance
Feeling that there is hope that the patient will get better.	Reassurance
Being sure that the best treatment possible is being given to the patient.	Reassurance
Receiving explanations that can be understood.	Reassurance
Feeling that the hospital staff is interested in the patient.	Reassurance
Knowing who can give the information that the family member needs.	Information
Knowing why certain treatments are being administered to the patient.	Information
Knowing who the professionals caring for the patient are.	Information
Being informed about possible transfers.	Accessibility
Being able to see the patient, frequently.	Accessibility
Feeling accepted by the hospital employees and staff.	Comfort
Knowing which other professionals can help.	Support

## DISCUSSION

The high prevalence of chronic patients that we found leads us to underline the importance of the involvement of members of inpatients’ families in their hospital care, independent of the severity of these patients’ clinical situation. The profile of members of their families was the same as described in other studies conducted in critical care units.^1-3,5^ In our study, as in studies cited previously, what stood out was the need for reassurance and information. These questions stood out regardless of the patients’ and family members’ characteristics. From the questions considered important by all family members, it could be seen that they wanted to receive information about the patient, in an open and understandable way that allowed them to feel sure that the patient was receiving the best treatment possible. They attempted to be vigilant about everything taking place in the hospital and regarding all the staff caring for the patient.

Members of patients’ families undergo a period of stress during hospitalizations and the first days are rife with doubts, anxiety and anguish.^1^ Few family members were able to state the names of the professionals who were caring for the patient. This could generate further stress, as this was pointed out as being a very important need. This lack of information causes significant harm to family members who need to get organized and understand hospital routines. This initial stress could be minimized if they were to receive some guidance during the first days of hospitalization.^5^

Our hypothesis was supported by the results from studies carried out in intensive care units but it was not confirmed. The members of the families of inpatients in a clinically more severe condition or presenting psychiatric comorbidity had the same needs as did members of the families of patients in a clinically less severe condition or without psychiatric comorbidity. Our supposition is that the experience of hospitalization, independent of patients’ characteristics, is what leads to such needs. This point deserves further studies to examine this situation in greater depth.

## CONCLUSIONS

The needs pointed out by members of the families of chronic patients hospitalized in general wards were similar to the needs pointed out by members of the families of acute patients hospitalized in intensive care units: they considered it to be very important to receive reassurance and information during the hospitalization process.
